# “Being seen” at the clinic: Zambian and South African health worker reflections on the relationship between health facility spatial organisation and items and HIV stigma in 21 health facilities, the HPTN 071 (PopART) study

**DOI:** 10.1016/j.healthplace.2018.11.006

**Published:** 2019-01

**Authors:** Virginia Bond, Sinazo Nomsenge, Monde Mwamba, Daniel Ziba, Alice Birch, Constance Mubekapi-Musadaidzwa, Nosivuyile Vanqa, Lario Viljoen, Triantafyllos Pliakas, Helen Ayles, James Hargreaves, Graeme Hoddinott, Anne Stangl, Janet Seeley, on behalf of the HPTN 071 (PopART) study team

**Affiliations:** a Zambart, School of Medicine, Ridgeway Campus, University of Zambia, Lusaka, Zambia; b Department of Global Health and Development, Faculty of Public Health and Policy, London School of Hygiene and Tropical Medicine, London, UK; c Desmond Tutu TB Centre, Department of Paediatrics and Child Health, Faculty of Medicine and Health Sciences, Stellenbosch University, K-Floor, Clinical Building, Tygerberg Medical Campus, Francie van Zyl Drive, Tygerberg 7505, South Africa; d Department of Social and Environmental Health Research, London School of Hygiene and Tropical Medicine, Keppel Street, London WC1E 7HT, UK; e Department of Clinical Research, Faculty of Infectious and Tropical Diseases, Keppel Street, London WC1E 7HT, UK; f International Center for Research on Women, Washington. D.C., USA

## Abstract

Health workers in 21 government health facilities in Zambia and South Africa linked spatial organisation of HIV services and material items signifying HIV-status (for example, coloured client cards) to the risk of People Living with HIV (PLHIV) ‘being seen’ or identified by others. Demarcated HIV services, distinctive client flow and associated-items were considered especially distinguishing. Strategies to circumvent any resulting stigma mostly involved PLHIV avoiding and/or reducing contact with services and health workers reducing visibility of PLHIV through alterations to structures, items and systems. HIV spatial organisation and item adjustments, enacting PLHIV-friendly policies and wider stigma reduction initiatives could combined reduce risks of identification and enhance the privacy of health facility space and diminish stigma.

## Highlights

• Spatial dimensions of stigma are linked to accessing HIV treatment in clinics.

• Distinct demarcation and client flow trigger visibility of People Living with HIV.

• Approaching HIV services carries a social risk of unwanted disclosure for PLHIV.

• Thoughtful spatial organisation and labelling reduces the chance of “being seen”.

## 1 Introduction

People living with HIV (PLHIV) often report experiencing a fear of “being seen by others” when in health facilities, and this can sometimes adversely affect engagement in care and adherence to anti-retroviral treatment (ART) (Gilbert and Walker, 2009, Horter et al., 2017, Raveis et al., 1998, Wringe et al., 2009). To reflect and address the relationship between health facilities and HIV stigma, we decided to focus on the role of the spatial organisation of health facilities, and HIV services within them, and on items linked to those HIV services. The spatial organisation of health facilities encompasses planning and layout of infrastructure including inter-spatial relationships within and between buildings and client flow systems. Items linked to HIV services arise from the health care and administrative needs of health delivery specific to a health condition and include copies of policies and guidelines, drugs, labelling techniques (for example, sign-posts, patient cards, folders, stickers, posters and numbering), client feedback mechanisms (for example, suggestion boxes), laboratory equipment and protective items (for example, gloves). The spatial organisation and items associated with HIV services can, by identifying PLHIV to others in health facility settings, enhance the risk of perceived and enacted forms of stigma. An example of this is shown in the work of Owolabi et al. (2012) in Nigeria who found that PLHIV in Nigeria worried that being seen collecting or carrying anti-retroviral drugs (ARVs) at the health facilities could lead to unwanted disclosure, and PLHIV experienced demarcated HIV services as a form of discrimination. Despite “being seen” in health facilities persisting in stigma literature (Gagnon, 2015, Li et al., 2007, Wolf et al., 2014), most stigma research in health facility settings has concentrated more on relations within the health facility between providers and clients (Andrewin and Chien, 2008, Famoroti et al., 2013, Li et al., 2007, Opollo and Gray, 2015, Stringer et al., 2016) and broader experiences of PLHIV (Gagnon, 2015, Horter et al., 2017) than on the physical environment (Gagnon, 2015, Li et al., 2007, Sullivan, 2012, Uebel et al., 2013).

Studies that have assessed stigma and the health facility environment have reported on policies, the availability of training on medical ethics and universal precautions, and availability of protective supplies to prevent workplace transmission (Nyblade et al., 2009, Owolabi et al., 2012). Additionally, interventions to reduce stigma in health facilities have focused mainly on shifting provider attitudes and changing institutional policies to create a more supportive environment for PLHIV seeking care (Nyblade et al., 2009, Stangl et al., 2013). For example, a “Person Living with HIV/AIDS-friendly achievement checklist”, originally developed in the context of three Indian hospitals, is intended to self-evaluate and improve services for PLHIV (Mahendra et al., 2007), and catalyse action (Nyblade et al., 2009).

Physical and built health environments represent an amalgamation of the different values and qualities of the broader society (Smyth, 2005), which are carried into the space by clients and health workers and are shaped by specific health conditions. This is illustrated in relation to HIV by the movement of PLHIV in a Californian prison (Stoller, 2003) and a Tanzanian hospital (Sullivan, 2012). In the Californian prison, the order and planning of confined spaces delayed access to care for a woman LHIV who was acutely ill, demonstrating the oppression of incarceration (Stoller, 2003). In Tanzania, newly built and well-resourced HIV services are a stark comparison with other hospital services demonstrating the inequities produced by global funding (Sullivan, 2012). Health facilities are thus an intersection of the physical environment, the values, attitudes and social interactions experienced in the place.

Historically, the development of HIV services has been accompanied by global funding and changes to spatial organisation including new infrastructure to deliver HIV services (Sullivan, 2012, Topp et al., 2012). Yet literature on the demarcation of HIV services shows a discord related to the impact of space demarcation on HIV stigma. It denotes both the perceived presence of stigma in certain parts of health facilities and the perceived absence of stigma in other places (Gagnon, 2015) and a contradiction in evidence about the possible risks and benefits of demarcating HIV services. The integration of HIV services and harmonised infrastructure in Zambia were, for example, shown to improve client experiences of accessing health services including reducing stigma (Topp et al., 2012) but in South Africa, other research on the integration of HIV care into primary health services illustrated that separate buildings for ART services were identified as stigmatising by clients and nurses (Uebel et al., 2013). To deepen the contradiction, one recorded benefit of demarcated HIV services has been the engendering of solidarity and a sense of community brought about by the sharing of HIV experiences within a particular space (Cataldo, 2008, Collins et al., 2016, Surlis and Hyde, 2001).

In addition to the demarcation of space, labelling techniques are used in health settings to distinguish between different clients and to align their needs to clinic personnel and procedures. Here evidence is not contradictory, with different forms of labelling being reported as highly stigmatizing and sometimes breaching basic standards of confidentiality for PLHIV. Studies in Dublin (Surlis and Hyde, 2001), Ethiopia and Tanzania (Nyblade et al., 2003) showed how PLHIV felt uncomfortable about certain material items (e.g. stickers, signs, use of gloves) that signalled their status. For example, PLHIV have reported hiding and/or removing ART from labelled drug packaging to avoid unwanted disclosure (Owolabi et al., 2012, Ware et al., 2006). Being identified as afflicted or different are central to the notion of stigma (Goffman, 1963).

The analysis in this paper documents how health workers in 21 health facilities across Zambia and South Africa (2015–16) recounted the relationship between health facility space, HIV services and HIV stigma. Health worker participants discussed their perceptions and experiences of HIV stigma linked to physical contact with health facilities and described what spatial and item adjustments were and could be made to reduce the risk of PLHIV identification. This enquiry builds on other evidence on HIV stigma in the same 21 communities and falls within research conducted as part of a wider community randomised trial, HPTN071 (PopART) (see, Hayes et al., 2014). In 2013, rapid qualitative research across all communities had identified a*”fear”* of or feeling *“shy”* about *“being seen”* accessing HIV services at clinics, observed Zambian PLHIV throwing ART packaging away, documented concerns that *“self-stigma”* put some people off going to HIV services at local clinics and noted the absence of stand-alone stigma reduction efforts at clinic and community level (Bond et al., 2016). In 2013–2014, 35.5% of a random sample of PLHIV (n = 3859) in all 21 communities reported some form of stigma (Hargreaves et al., 2018). A nested case control study of PLHIV further revealed that feeling ashamed about having HIV contributed to delayed linkage to care (Sabapathy et al., 2017) and other qualitative research flagged HIV stigma (including the risk of ‘being seen’) as a key factor (Seeley et al., 2018). Combined, data specific to these 21 communities reveal stigma as a persisting barrier to seeking care, also evident from other studies in the region (Hasan et al., 2012, Raveis et al., 1998, Sabapathy et al., 2017, Wringe et al., 2009). Not only does this affect individual health outcomes, it also hinders global aims of addressing the HIV epidemic.

## 2 Methods

### 2.1 Study design

Qualitative research was undertaken as a component of an ancillary study of HIV stigma experiences amongst health workers in 21 urban communities in Zambia and South Africa (Hargreaves et al., 2016). The stigma ancillary study is nested within HPTN071 (PopART) trial, which aimed to reduce HIV incidence through a combination HIV prevention approach (Hayes et al., 2014). Community health workers spearheaded the intervention by regularly visiting households for three years (2014–17), encouraging home-based HIV testing, screening for tuberculosis and sexually transmitted infections and linking newly diagnosed PLHIV to treatment at the main government health facility (Hayes et al., 2014). From August 2015 to May 2016, this qualitative study was conducted following baseline studies (Bond et al., 2016; Hargreaves et al., 2018) and the first year of the HPTN071 (PopART) study intervention in one health facility in each community. The study aim was to understand how the organisation of HIV services within these facilities and other HIV items might influence HIV stigma.

Study participants (aged 18 and above) were recruited in each facility from three categories of health workers, namely: government health facility workers (HFWs), community health workers (CHWs) and HPTN 071 (PopART) specific community health workers referred to as Community HIV care Providers (CHiPs) (see Table 1 and Bond et al., 2016). The distinction between these two types of community workers is important for this analysis since the interactions and affiliations with the health facilities were different and this may have affected their feelings and experiences of health facility space and stigma. CHWs were more embedded in the health facilities since they were affiliated with and located within health facilities and their presence pre-dated the HPTN 071 (PopART) intervention. Whereas CHiPs were affiliated to the trial, located within the community and referred clients to the health facility and were only present in the 14 of the 21 communities that were part of the intervention (see Hayes et al., 2014).

**Table 1:** Profile of health worker participants.

		**Zambia**		**South Africa**		**Total**
	**Site**	**Intervention sites**	**Control sites**	**Intervention sites**	**Control sites**	
	**Participants**	45	21	33	15	114
**Type**	**HFW**	15	13	12	9	49
	**CHiPs**	14	0	16	0	30
	**CHW**	16	8	5	6	35
**Gender**	**Women**	30	19	26	14	89
	**Men**	15	2	7	1	25
**Age**	**20–39**	15	2	21	8	46
	**40–59**	12	8	11	7	38
	**60+**	4	1	0	0	5
	**Age unknown**	14	10	1	0	25

### 2.2 Data collection

**Fig. 1:**
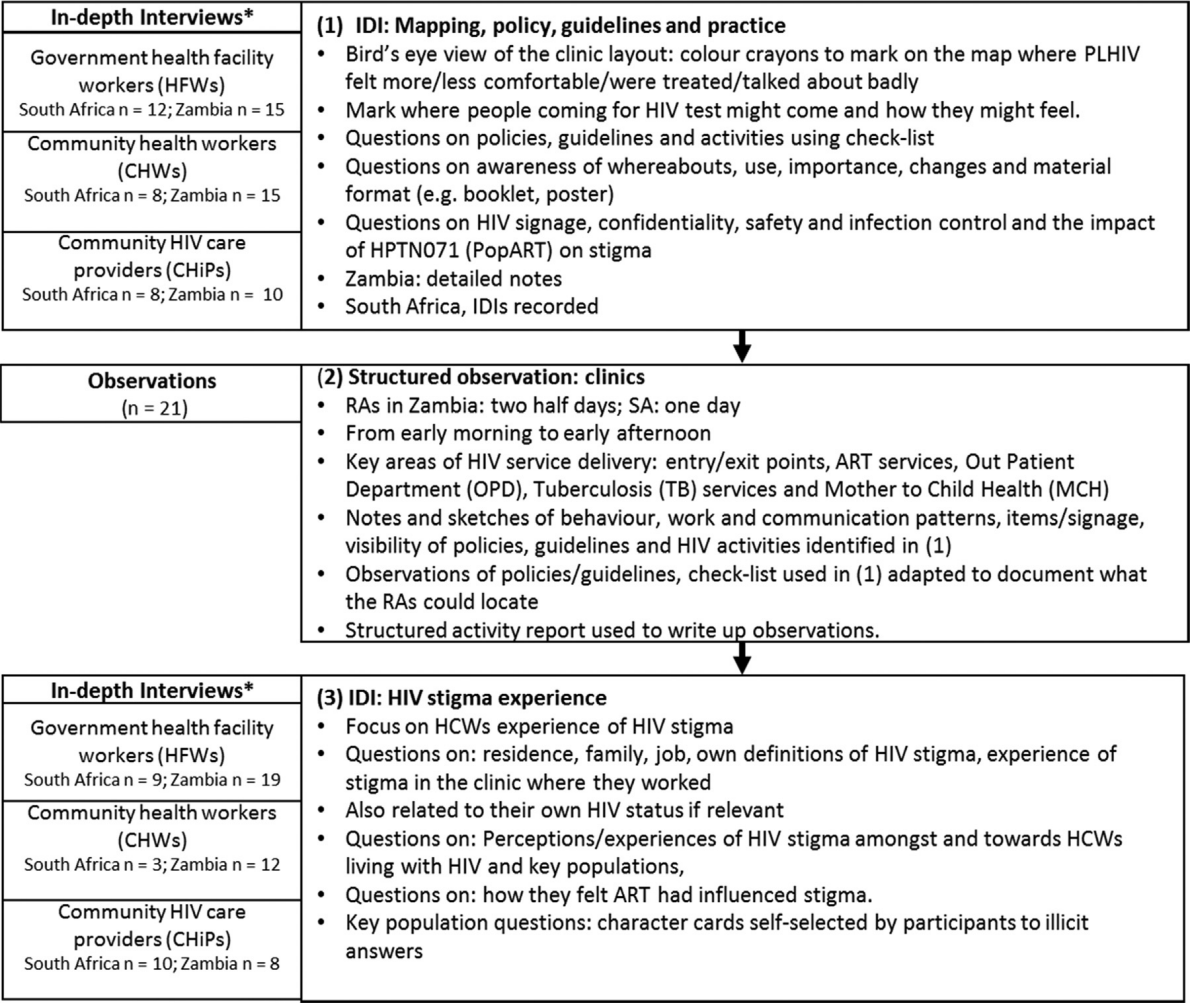
Sequence and Detail of Research Methods and Tools. *Note: In South Africa, two health workers participated in both the mapping and the HIV stigma experience discussion. In Zambia, 13 health workers participated in both discussions.

Three research tools were used in sequence (see Fig. 1 below). We began with an in-depth interview using a map of the health facility to explore participant perceptions and experiences about how comfortable and uncomfortable PLHIV feel in different places within the respective facilities, and whether PLHIV were ever talked about badly in these places. The choice of “comfort/discomfort” was to allow participants to talk about how these places “feel” for PLHIV rather than ask about stigma directly. The terms “comfort/discomfort” are also sometimes used in stigma indicators (Wouters et al., 2017). In addition, using an adaptation of the People Living with HIV/AIDS-friendly achievement checklist (Mahendra et al., 2007) and health facility audit tools (Topp et al., 2012), participants were asked about different policies, guidelines and about HIV signage, confidentiality, safety and infection control, as well as the impact of HPTN 071 (PopART) on stigma. Following a review of these mapping IDIs, structured observations were carried out by research assistants in each of the health facilities using an activity report form. Once observations were complete, a second set of in-depth interviews with health workers explored their experience of HIV stigma.

### 2.3 Study population

For the first interview, research assistants, assisted by local study teams (including the first and second authors who conducted some interviews), identified and approached at least one participant from each health worker category who were: willing to be interviewed, considered qualitatively representative of their group, not new to their job or the clinic, and who had participated in the baseline stigma survey and said they were willing to participate in subsequent qualitative research. A mix of age and gender from each facility was desirable but not always achieved, in part because most health workers are women (Hargreaves et al., 2018). For the second interviews, one HFW who was key to the delivery of ART (for example, in Zambia, a sister-in-charge of ART) and/or Health Workers who had shared they were living with HIV, and one CHW and CHiP, who had participated in the baseline survey and who were willing to be involved in qualitative research were identified and approached. A total of 49 HFWs, 35 CHWs and 30 CHiPs participated in the study, representing at least three health workers in each facility. Amongst the total, 37 self-identified as living with HIV across both interviews; 27 in Zambia and 10 in South Africa.

### 2.4 Data analysis

At an initial analysis workshop in May 2016, transcribed and summarised interviews, observations and map data from each community were read and reviewed separately by three members of the research team (including first and second authors). A broad set of thematic groups of data were identified in collective discussions. These thematic groupings were then used as a frame through which the findings were processed further for a deeper understanding of the messages embedded within it. This resulted in five aspects of the organisation of health facility space emerging as contributing to both anticipated and enacted HIV-related stigma. These were: physical infrastructure, material items, client flow, relations within the facility and the personal journeys and social identities of PLHIV.

At a second analysis workshop (June 2017), the research team revisited and refined the themes and developed a coding framework that included the five aspects earlier identified as well demarcated areas within the clinics (for example, the pharmacy), experiences of stigma (anticipated, enacted, internalised), management of stigma, policies/guidelines/training and the influences of HPTN 071 (PopART) on stigma. All data in text form were then managed and coded using ATLAS.ti (Scientific Software Development GmbH). Subsequent code reports were then analysed and written up as thematic summaries.

**Fig. 2:**
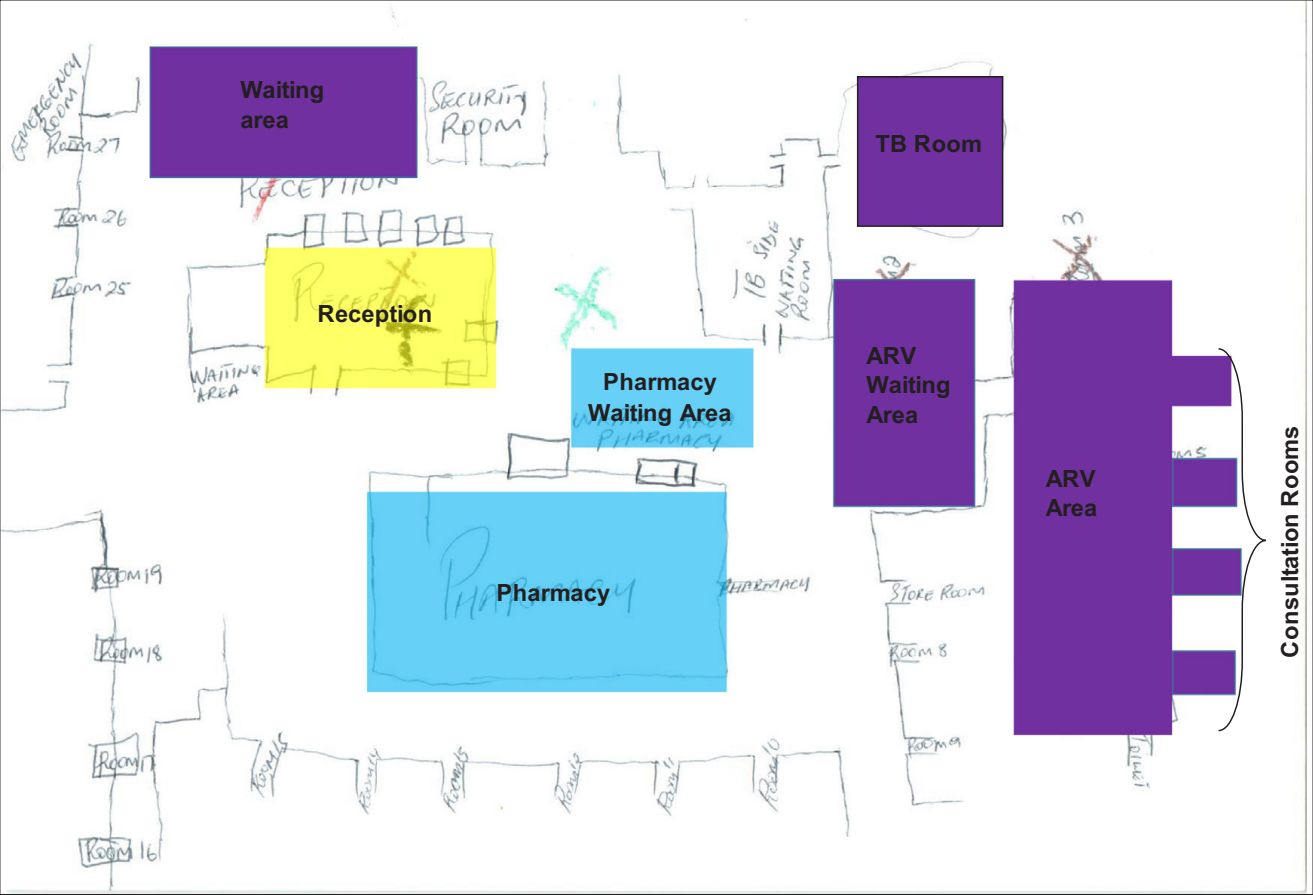
SA Map: Participants assessment of comfort, discomfort, being talked badly about and location of HIV testing. **Key**:- Blue: Comfortable, Purple: Mixed outcome – comfortable and uncomfortable, Yellow: Location of HIV Testing, ART/ARV: Antiretroviral (department), MCH: Mother & Child Department, OPD: Out-Patient Department.

**Fig. 3:**
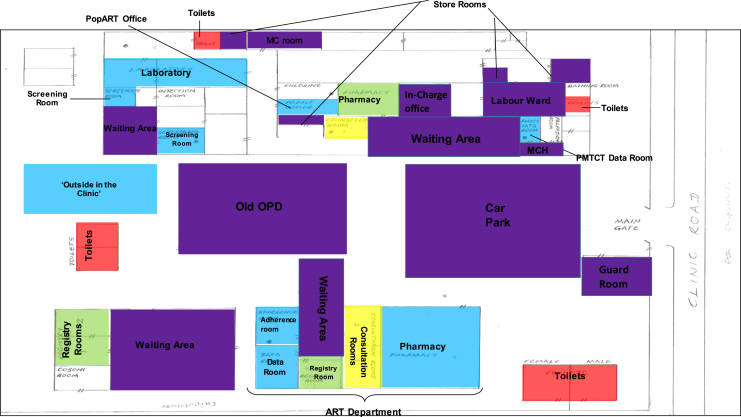
Zambia Map: Participants assessment of comfort, discomfort, being talked badly about and location of HIV testing. **Key**:- Blue: Comfortable, Green: Uncomfortable, Purple: Mixed outcome – comfortable and uncomfortable, Red: People Spoken about Badly, Yellow: Location of HIV Testing, ART/ARV: Antiretroviral (department), MCH: Mother & Child Department, OPD: Out-Patient Department.

Data not in text form, specifically the maps (n = 68) and observation checklists (n = 21), were systematically and manually analysed. The maps were each scanned and analysed manually by moving between the individual colour coding of maps (see Fig. 2) and reviewing the corresponding interviews where participants explained why they felt a particular way about a certain part of the health facility. For example, why the ART department was coded as uncomfortable/comfortable/somewhere people talked badly about PLHIV. This information was extracted into an Excel spreadsheet in a two-step process. Firstly, for each country and facility (and corresponding participants), separate sheets captured explanations about comfort, discomfort, talked badly about and HIV testing. Secondly, drawing on the latter process, a series of summary matrices for each country presented for each area of the health facility (for example, ART waiting area) how many (and why) participants identified particular areas as aligning mostly with a mixed outcome of comfort/discomfort or mostly comfortable/uncomfortable/talked badly about. Sometimes participants would determine these spaces to be one or the other, and some participants would explain they could be both. This allowed us to then develop the generic maps for each country reflecting the pattern that emerged across all the maps and interviews (see Fig. 2, Fig. 3). The outcome in the maps was reached by assessing the general feeling about a particular area based on how many participants explained their experience. For example, in South Africa, the ART waiting area was experienced being both comfortable and uncomfortable (see Fig. 2); and in Zambia, the pharmacy in the general area of the health facility was experienced by most participants as uncomfortable (see Fig. 3).

The observation checklists topics were also collapsed into a country specific matrix that captured for each facility the visibility, location and format of each policy and guideline. From individual interviews, corresponding detail on awareness, the use and importance of each was also entered in the matrix. For the analysis we then worked across these different forms of data.

### 2.5 Procedures and ethics

Prior ethical approval for all study procedures was obtained from the institutional review of the London School of Hygiene and Tropical Medicine (LSHTM), the Health Research Ethics Committee at Stellenbosch University, and the Bio-medical Ethics Committee of the University of Zambia. In-country teams managed the data. All participants were informed during a written informed consent process that the researchers were also interested in the experiences of health workers living with HIV. During the interview process, participants were then asked if they were willing to disclose their HIV status. Incentives were not given due to their potential to adversely impact the stigma survey and wider trial, which involved large numbers of clients and participants.

### 2.6 Findings

We begin the presentation of our findings by focusing on the spatial organisation and items of HIV services. We then present Health Workers experiences of how space is navigated by PLHIV and how material items can signify HIV status. In the health facilities, the physical layout and infrastructure, client flow systems, demarcated services, items and the visibility of policies and guidelines had the potential to interact with HIV-related stigma. We use the term ‘clinic’ for ‘health facilities’ in the following text as this term was used by participants.

### 2.7 Physical layout of clinics

The 21 clinics were all included in the HPTN 071 (PopART) trial and had HIV services including providing anti-retroviral treatment (ART). The population catchment for each clinic varied from 14,500 to 161,615. The clinics are in high density housing areas and fenced in. In Zambia, there is usually one entrance whereas in South Africa, there are two entrances (one for pedestrians and one for vehicles). Security measures are more evident in South Africa but in both countries, guards control access and parking. The clinic buildings are owned and mostly built by the government and share core design features. The lay-out is typically organised around basic services. Out-patient department (OPD), pharmacy, laboratory, maternal and child health (MCH) and staff rooms are typically in the main structure, and toilets, TB and HIV services are in either separate single storey infrastructure or demarcated areas. In Zambia, mortuary services exist in separate buildings. Car parking and small garden areas are located within the boundary fence or wall. There is limited room for expansion. No South African clinics had in-patient wards, whereas in Zambia all the clinics had maternity wards and some also had female and male in-patient wards.

In Zambia, at the time of the study, the ART department (often called *“the ART block”*) was in a separate concrete building in 11 of the 14 clinics. In two clinics, the ART department was visible from the gate and in the others, the ART department was around the back of the site, behind the main clinic buildings. In South Africa, the ART department was located either at the periphery of the clinic (n = 3) or in a separate pre-fabricated structure outside the clinic building (n = 6) referred to as a *“bungalow”*. Three of the latter bungalows were in full view of the main entrance and three were behind the main clinic.

The HPTN 071 (PopART) trial had provided resources to health service partners to optimise HIV services in all the 21 health facilities. Some of these resources had been used to add either temporary or permanent structures related to HIV services to the health facilities. In Zambia, these included information desks (consisting of a desk and a small marquee or tent) near the entrance of the eight trial intervention clinics, and then according to the needs of each clinic, some also had metal containers for bulk drug storage (n = 6), permanent concrete shelters for the ART department clients (n = 6), and pre-fab containers for trial staff and files (n = 2). In South Africa, these additions included two ART structures.

### 2.8 HIV client flow

**Fig. 4:**
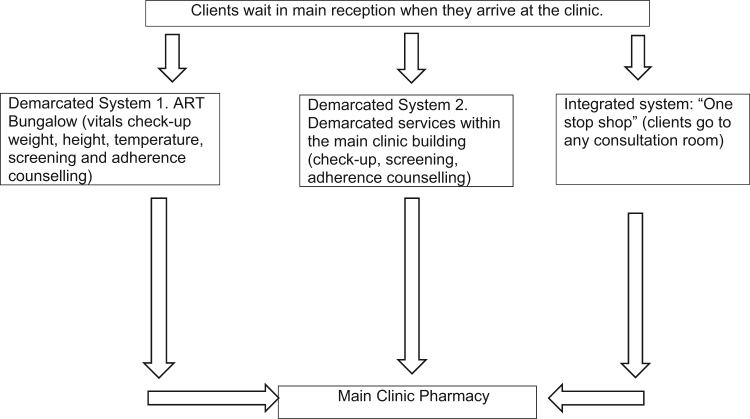
HIV client flow chart in South African clinics.

**Fig. 5:**
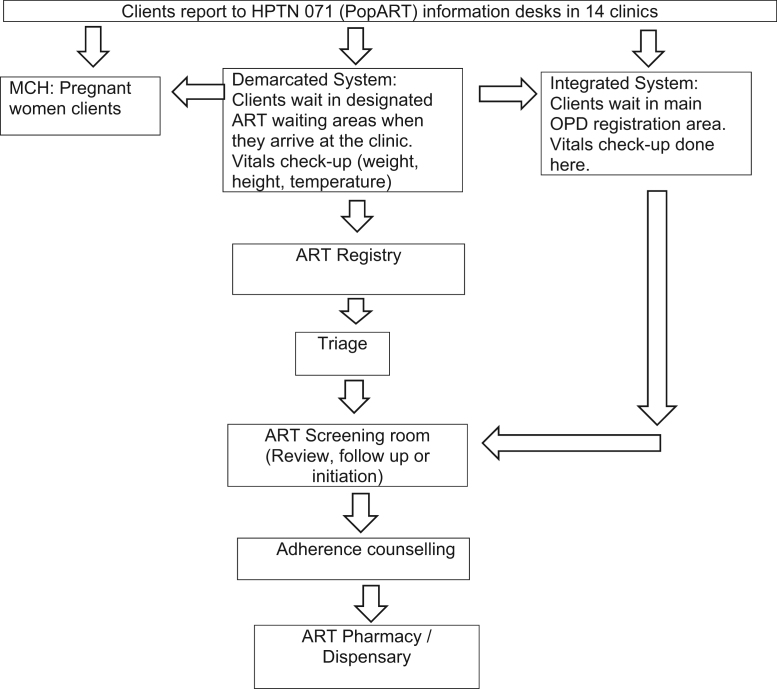
HIV client flow chart in Zambian clinics.

For an individual PLHIV accessing HIV services involves a distinctive movement through interconnected and often demarcated places on a relatively frequent (approximately every three months) basis, as summarised in Fig. 4, Fig. 5. As reflected in Fig. 5, the HPTN 071 (PopART) trial in the 14 intervention communities reconfigured client flow processes, particularly in Zambia with the introduction of information desks located near the clinic entrance. In both countries, HIV service staff numbers were increased, and their presence also had an impact on client flow. For example, in South Africa, extra counsellors were available in the pre-fabricated ART structures.

The client flow for PLHIV differs across countries and clinics. In both countries and all clinics there was an appointment and queue management system for PLHIV. In Zambia, certain days were also sometimes reserved for adolescents and children and HIV laboratory tests. In South Africa (see Fig. 4), all PLHIV clients were first filtered through the main reception alongside all other clients. In one clinic, there was a specific window at reception reserved for PLHIV. From the main reception, there were three different flow systems in place across the nine clinics which involved PLHIV either going to an ART bungalow or to demarcated services within the main clinic building or to any consultation room. The latter flow system was referred to as a “one-stop-shop” model where all nurses were licensed to care for and administer drugs to PLHIV. In all South African clinics, PLHIV would collect their medication from the main clinic pharmacy unless they were part of an ART club. In two clinics, PLHIV who were part of ART clubs had their medication pre-packed and distributed to them through the club network, with individual PLHIV taking it in turns to collect medication for other members of their club.

In most of the clinics in Zambia (see Fig. 4 and Simuyaba et al., 2017), PLHIV clients started their appointment day by waiting outside the demarcated ART department and were often recipients of a health talk given by a CHW. Once inside the ART department, they moved through a sequence of spaces for triage, screening, adherence counselling and the pharmacy. PLHIV only went to the main clinic in this flow system if they were referred to the laboratory or other services, except for one clinic where a specific pharmacy window in the main clinic was where ART clients received their medication. Another flow system operated in one Zambian clinic and was referred to as an ‘integrated system’. In that clinic PLHIV would start with other clients in the main waiting area and be filtered from there to demarcated HIV services located within the main clinic building. In all clinics, pregnant women LHIV accessed consultation and medication from demarcated MCH services until their child was two years old. They were often asked to wait until all other MCH activities were completed and to only attend MCH on specific days.

Client flow either heightened or lessened the identification of PLHIV. Concerns about being identified moving through the clinic system are captured in the following description:

*“…they [PLHIV] will hesitate to go to the ART clinic and they will look at the direction of the OPD to see if there is no one seeing them. That is when they would go to the ART [clinic] and sometimes when they are at the ART pharmacy area they might feel uncomfortable because they fear that when they see them collecting ARVs, they think their neighbour might tell others that they saw him or her at the ART [clinic] where they collect ARVs” (CHW_W_61_Z).*^1^

When PLHIV were filtered through a general waiting area and/or registry, common in all South African clinics and one Zambian clinic, the chances of being identified diminished: “*At the OPD, registry and triage patients are comfortable because the system is integrated. No one would know who is HIV positive and not”* (CHiP_W_41_Z). Another health worker in the same Zambian clinic felt that not having distinctive HIV services was helpful:

*“We have incorporated everyone like, whether the person is HIV positive. The person is negative they collect the drugs at the same point. The same pharmacy. They are seen by same clinicians. Yah, so there is nothing like no this one is HIV positive, they have got their own room or what” (HFW_W_Z).*

However, overall (with some exceptions) there were mixed feelings about how PLHIV experienced general waiting areas, entrances, car-parks and departments not demarcated for HIV, as reflected in both maps (see Fig. 2, Fig. 3). Across participants and countries, most spaces where PLHIV were alongside other clients were deemed to be both more and less comfortable and were also the spaces where participants said that PLHIV were the most likely to be talked badly about. In relation to being comfortable, participants echoed the sentiments of the integrated clinic system and said it was harder to identify PLHIV in general waiting areas (including OPD in Zambia), which are shared by clients with different needs and ailments. As one South African participant explained, *“Everyone has to pass through the reception area and therefore you cannot attach an ailment to this space”* (HCW_F_23_S). But these general spaces were also said to be uncomfortable. This was partly attributed to PLHIV feeling more worried about *“sticking out”* or being *“singled out”* when surrounded by other clients and this, according to participants, was a form of *“self-stigma”*. Additionally, nurses asking clients questions in open and non-ART demarcated spaces about HIV status, CD4 count and aspects of treatment, and calling out of names waiting areas had the potential to negate confidentiality and foster feelings of discomfort for PLHIV. Participants also said that *“normal”* health workers and clients *“with other diseases”* were more likely to gossip or talk badly about PLHIV in these spaces. According to participants, these were more common if PLHIV were visibly ill, linked to an item associated with HIV (such as a particular type of clinic card) or publicly asked a questioned about their status. As one Zambian participant commented about PLHIV in OPD, *“People will notice if you are very ill and say you should be in ART - people will tell you 'you can’t get ARVs here'”* (CHiP_M_69_Z). And a South African participant who marked the general waiting area as uncomfortable explained, *“The colour coded clinic cards makes PLHIV feel uncomfortable because it identifies them as HIV positive to the public”* (CHW_LWH_F_25_S).

The main clinic pharmacy was regarded overall as more uncomfortable in Zambia for PLHIV because of being too open, having long queues and PLHIV being seen collecting ART in *“boxes”.* This compared to South Africa where the pharmacy was assessed as more comfortable because anyone with a prescription would go there and as such, no one could distinguish PLHIV from others. In Zambia, the laboratory was identified as being overall comfortable for the same reason that HIV status is less obvious since every client is *“in a similar position”* and involved in having a test. However, in one health facility, a health worker described how particular week days were allocated to only do HIV tests in the laboratory and on this day, for PLHIV the laboratory waiting area became uncomfortable.

Thus, alongside flow, other features were aligned by participants as facilitating identification including demarcation, long waiting times, items and other visible features.

### 2.9 Demarcation of HIV services

Clinics, as demonstrated above, enact delivery of care through demarcations. Demarcation was often spoken about as *“separate”*. Several *“separate”* HIV service spaces are associated both with feelings of comfort and discomfort. ARV waiting areas, for example, are assessed as inciting both comfort and discomfort in both countries. They facilitate interaction between PLHIV and provide privacy and specialised staff, yet the long queues and waiting times increase the chance of “being seen”. Further, ARV waiting areas that were easily visible to others or conversely more hidden from others, affected feelings of comfort. This common pattern across both countries is captured by a Zambian CHW participant who reflected about the ARV waiting area that on the one hand: *“People feel free because they know that there are people whom they share the same situation with. The place is hidden”*, and on the other, *“…people who come to ART for first time do not feel comfortable because they fear to be seen by neighbours and they do not know what to expect. People from lab and OPD might see them”* (CHW_W_61_Z).

As already inferred, spatial organisation made PLHIV visible. Zambian participants mentioned how the direction people faced when seated on benches in the ART department and the proximity and orientation of the ART waiting area to the clinic's main entrance can bring about social discomfort for PLHIV if either is clearly visible from the entrance. Similarly, in South Africa, because some ART bungalows are located behind the clinic's main building, once a person is seen going around the back of the clinic building, they will be assumed to be living with HIV. A South African participant mentioned that clients should avoid a particular passage located near the ART consultation room and rather make use of a back door.

Conversely, separate rooms, walls and restricted entry facilitated privacy. In Zambia, once PLHIV were within the ART department and could not be seen, it became more comfortable. One CHiP referring to the position of an ART consultation room, *“because they [PLHIV] are alone with the health care workers so no one will hear what they are saying and also the person they will be talking to will be focused on them and their wellbeing”* (CHiP_W_27_S). Privacy was often linked to feelings of comfort, safety and confidentiality and one on one discussions with health workers. Parts of the clinic identified by participants as providing a level of privacy that secured confidentiality included consultation rooms in both countries and MCH, a nutrition room and adherence counselling rooms in Zambia. In one Zambian clinic, the laboratory was also *“perceived [as] the place to be confidential as it was located in a private place and the entry was restricted”* (observation notes, Z).

Related to the concept of privacy is the idea of communality and social cohesion in places like the “ART bungalow”, rooms used for alternative distribution of ART through adherence club systems (available to “stable” clients to make monthly ART collection simpler) and ART waiting areas. In South Africa, for example, PLHIV are said to be comfortable within ART service places because they can build rapport with one other and with health workers, give and receive support and share their experiences of living with HIV. A community health worker in Zambia corroborated this: “*The establishment of [the] ART block has made people living with HIV comfortable because they don’t have to mix with other clients who have come for different services like malaria*” (CLW_W_71_Z).

There was no distinct variance in how health workers living with HIV and those not living with HIV categorised space according to feelings of comfort and discomfort. It was notable that health workers living with HIV did not often bring out their own experience but rather tended to talk about the experience of other PLHIV in these spaces, reflecting a general reluctance to discuss their own HIV-status. However, there were a few who were more sensitive to any segregation of PLHIV within the clinic. Some also relayed the challenges that emerge from being a healthcare worker and LWH by reflecting on how these characteristics interface with spatial organisation of health facilities, as illustrated by one SA CHiP living with HIV.

“*People know that there is a specific door that is used only by people who are there to fetch their ARVs and even us people who are already in clubs, people know that when we go into that bungalow that we are sick and that all the people that are in clubs are sick…I have a problem with my job. I can’t lie… my only problem is with the people here at work and also in the community because it doesn’t feel good to know that in the community people are always discussing your health…if someone sees me in the clinic and they ask me why I am at the clinic I tell them that I am there for the same reason as them…. Even at work, I have a problem because people don’t treat you or see you in the same way…. Although we are healthcare workers and we can preach confidentiality, people still gossip”* (CHiP_ LWH_W_S).

Although some health workers in both countries considered demarcation to be more efficient and a way of making clients more comfortable, most of them also regarded it as a potential trigger for assumptions that any clients seen approaching or in the demarcated HIV service areas must be living with HIV. According to a South African worker (CHiP):

*“Another thing that causes people not to go for treatment at the clinic is that there is one clinic building and then behind the clinic there is another one designated to people who have TB and those who are HIV positive. So, no matter what I have gone to the clinic for, as soon as I go around to the back, people already look at me and conclude about me”* (CHiP_M_25_S).

Zambian participants were less critical of demarcation than those in South Africa. South African participants criticised and condemned demarcation, associating it with PLHIV experiencing stigma and discomfort. A community health worker, for example noted that:

*“The way I understand it, I would say that stigma is…for example people are separated. There are those in the TB area and there are those in the ARV section. I would say that stigma applies to those who are in the ARV section because everyone will now look at them differently because they have the own separate section”* (CHW_W_50_S).

For another South African participant, the demarcation of clients with certain conditions to particular places was equivalent to discrimination*: “At this clinic PLHIV sit on their own. People on TB treatment sit on their own. Sick people are on their own. So, it's like there is discrimination”* (CHW_W_29_S).

### 2.10 Long waiting times and congestion

*“Queues”, “overcrowding”* and *“long waiting time*” in compressed places (for example, corridors) and where space was limited increased the possibility of “being seen” and was closely associated by participants with congestion. Extended waiting times heightened the risks of being identified as living with HIV(based on where one is seen or what identifying items one might carry, and thereby exacerbated pre-existing discomfort and feelings of insecurity in the clinic).

### 2.11 Material Items signify HIV

Items specific to HIV can, with client flow, demarcation and long waiting times, not only place PLHIV at risk of unwanted disclosure but also reinforce PLHIV as being different from others.

*“If we come to the clinic together and we are here for something else and I am here for ART. Our cards aren’t the same. My card is green, yours is white. Within the clinic, I have my own side where I submit my card and you have your own place where you submit your card. Even our waiting areas are separate. You sit in another section and I know that I sit in this section. When I leave the clinic, let's say for instance I came to fetch my medicine package, I have this container with my medication for the month, whereas you just have pills in small plastic bags. There is a lot that can cause stigma….” (CHW_W_54_S)*

Participants seemed to agree that birth/death certificates, lab forms, x-ray forms, discharge sheets, beds and wards would not indicate HIV status. Signs around the clinics were not directly linked to stigma by participants. But referral slips, colours, client files/folders and/or cards and ARV packaging were material items that identified that any client was LHIV, particularly in Zambia. It was also said that PLHIV may feel uncomfortable in areas that had many voluntary counselling and testing (VCT) posters.

Zambian clinic and HPTN 071 (PopART) systems meant that PLHIV, after diagnosis, would be given a referral slip to access care. Characteristics of a referral slip (particularly the colour for example, [in this case yellow]) were a source of discomfort for many PLHIV. Some would fold and hide their referral slip (sometimes following the advice of CHWs or CHiPs to do this). However, a few participants said that referral slips were a key part of HIV diagnosis and if you felt comfortable about your status, you would not have this concern, as explained by a CHW, *“That would help me know my stand, that I am HIV positive or HIV negative.”* (CHW_M_69_Z).

In both countries, client files (or “folders”) sometimes could be linked to HIV status and had the potential to identify PLHIV to others. In Zambian clinics files were managed differently in different places. Sometimes only the ART department used files for clients or PLHIV files were a different colour or PLHIV files were stored in a different place. Some participants felt that HIV status could not be identified through files. Most participants felt that files and records were stored securely maintaining confidentiality, although in two Zambian clinics there were concerns about cleaners having inappropriate access to client files and cabinets with files not being locked.

In South Africa, “green clinic cards” indicated HIV status and PLHIV were reported to sometimes hide their cards, because they were uncomfortable holding the card. In one clinic, these cards were also placed in a special box, which was not done for any other client group.

One record type that was specifically mentioned in Zambia as indicating HIV status was “Under 5 Cards”. If you could interpret an “under-5″ card, you would be able to identify if the child and mother were living with HIV. Similarly, antenatal care (ANC) cards carried a veiled indication of HIV status as reflected in an observer report: *“On ANC cards there is something written for pregnant women who are HIV positive but only a health care worker can know, especially those trained on PMTCT” (P3_MCH_ Observations – 3.3-Zambia)*

Participants, especially in Zambia, often raised the potential of ARV packaging identifying PLHIV. Even the sound of ARV boxes in clothing or bags was said to expose PLHIV. An HFW explained what many PLHIV do after collecting their ART:

“We find empty bottles of medicines thrown away. Patients would rather put the drugs in their bags without their actual containers because they feel people will know they went to collect their drugs…three quarters you will find that they go into private rooms to remove the drugs from their containers…others remove their drugs [to put] in plastic bags, for the fear that maybe even family members might see the bottle” (HFW_W_39_Z)

### 2.12 Visibility of policies, guidelines and client suggestion boxes

Policies, guidelines and complaint processes (such as suggestion boxes) are designed to provide confidentiality and safety for PLHIV and health workers (both those LHIV and to protect them from HIV). The structured observations used to assess the presence of policies, guidelines and complaint processes established that policies and guidelines were most often in the form of posters and *‘books’* (especially in Zambia). Overall, participant awareness and use of a certain policy/activity/guideline often did not match with its existence, format or location, particularly in Zambia.

There were 56 instances in Zambia compared to just four in South Africa where one of the 11 policy/activity/guideline(s) were not seen in use in a clinic at all by observers; South African health workers have a much more direct access to policies and guidelines than in Zambia. Across both countries, participants were mostly aware of “Patient Confidentiality”, and “Testing and Counselling” policies, and least aware of, “Special services for health workers and community health workers living with HIV”, “Right to Health Care”, “Infection Control” and “Guidelines for managing HIV in the workplace”.

The potential link between the visibility and application of guidelines is exhibited in the following participant explanations, the first from Zambia and the second from South Africa:

*“Yes, I am aware of it, we were taught at the trainings which we underwent for psychosocial counselling and you may find posters about it at VCT [voluntary HIV counselling and testing], it is important for people because it makes them to feel comfortable and open up” (CHiP_W_35_Z).*

*“We have to, as a clinic, put posters and everyone's rights on the poster so that people can see that if you want to do something, go and do it outside the facility because we are against what you are doing. We do have posters especially inside the facility”* (HFW_W_23_S).

In all clinics in South Africa and most clinics in Zambia, suggestion boxes for complaints were known about by participants and located visibly, but in South Africa there was more observed use of the boxes by clients and staff.

### 2.13 The Clinic Space: navigating assumptions and relations within

Being physically present in health facilities combines with the social identity of PLHIV using services to influence how clinic space is experienced by PLHIV. Participant explanations reflected how being seen in the clinic combines with assumptions about HIV acquisition, and how relations within the clinic can appease or aggravate the situation.

### 2.14 Social assumptions about “being there”

The problem with “being seen” is that assumptions are made based merely on “being there”, as explained by a CHiP:

*“Even if it is someone who isn’t on treatment, who is there for something else, they will see a person, they will talk and they will put stains on a person. That's how people are. They see you in that place, they already have answers of why you are sitting in that place” (CHiP_W_25_S).*

Assumptions are made both about having HIV and how HIV was acquired. As one South African participant explained, someone who knows you *“knows what you are there for”* (CHW_F_41_S), and another commented how people who know you *“jump to all sorts of conclusions” (CHiP_W_33_S)*. Assumptions are made about having sex *“too early”* (if younger), *“without a condom”* or with *“so many”* partners or having HIV, *“out of their own will”*. Two South African participants (CHW_W_44_S; HFW_M_50_S) pointed out that it is your *“dignity”* that is being questioned and compromised. In both countries, discomfort with being seen by others in the clinic was said to be most pronounced when PLHIV were closer to diagnosis and newer to treatment and when visibly sick.

### 2.15 Navigating relationships in clinic spaces

Participants across all health worker cadres and in both countries in this study reported receiving both friendly, respectful and supportive treatment as well as unfriendly and intolerant treatment from Health Workers. These experiences, and anticipation of such, were at times associated with different clinic spaces.

Relations between nurses and other staff in HIV demarcated spaces was usually described as “friendly” and supportive. In these spaces, PLHIV who had been on treatment for longer sometimes took on an advocacy role and talked to new clients about being on treatment. Other than HIV services, the prep/observation room in South Africa and the nutrition and adherence counselling rooms in Zambian facilities, were reported to be comfortable spaces demonstrating friendliness, openness and confidentiality. Similarly, consultation rooms were valued for providing privacy for nurses and clinicians to be alone with clients and communicate confidentially. As noted earlier, in other more generalised and open services and areas, relations between PLHIV and health workers could be more strained, with health workers being less discrete about what they said about PLHIV. According to two participants (CHW_W_44_Z and HFW_W_57_Z) and observations, the labour ward and the maternity ward in Zambia were spaces where nurses can disclose or gossip about a person's HIV status.

### 2.16 Reconfiguring clinic space and stigma

If HIV stigma in the clinic was either anticipated or experienced, participants said that stigma was more likely to be circumvented than contested. Circumvention often involved reducing contact with clinics and/or the possibility of “being seen”. Discussions of stigma that might be faced by groups such as women involved in sex work or men who have sex with men also often highlighted the possibility of reconfiguring spatial organisation and items to reduce experiences of stigma.

### 2.17 Circumventing stigma

Confronted with the possibility of stigma, avoiding and reducing contact with clinic services was a common strategy. One participant said that even on the way to the clinic, PLHIV could decide to *“divert”* to escape being seen. Avoiding getting tested or treated at the nearest clinic completely and going either elsewhere (where the chance of being recognised was less) or giving the wrong home address to the clinic were common examples of tactics used. Sex workers, men who have sex with men, migrants, pregnant women, men and health workers living with HIV were identified as more likely to avoid services for as long as possible. For example, pregnant women living with HIV were said to often present late at ANC services to avoid the potential exposure of being seen accessing HIV services and extended waiting times. A South African CHiP relayed how a woman living with HIV refused to bring her child living with HIV to the clinic, *“because people will ask her why her child is sitting in a specific area whereas other children are sitting in another area”* (CHW_W_50_S).

Coming early or late in the day was another pragmatic strategy, or *“hiding”* in other places in the clinic or outside the clinic. Some Zambian health workers living with HIV said they would collect their own specimen or file and pretend it belonged to a client. Health workers living with HIV, people living with disabilities (PWD) and high-profile clients (for example, politicians and police) would use their status and/or presence to sometimes obtain preferential treatment. They would contact health workers who knew them (or felt sorry for them) to facilitate a more *“express service”* so less time was spent at the clinic. This sometimes took the form of health workers collecting drugs for them and *“meeting with that somebody somewhere”*. In Zambia this arrangement was referred to as a *“VIP”* service and could involve informal payments to health workers. Hiding ARVs was another avoidance strategy. Many clinics in Zambia provided bins located close to the ART pharmacy specifically so clients could throw away drug packaging.

Changes to items were sometimes suggested as a way of managing the risk of identification. A few South African participants and Zambian CHiPs suggested changing the colour of referral slips, folders and cards, as mentioned by one HFW, “*ARV patients have green cards, chronic patients have yellow cards. So, HIV is also chronic. Why not change the colour of the card to yellow?”* (HFW_W_23_S). One form of labelling in South Africa conversely facilitated less waiting time at the clinic. In some South African clinics, PLHIV belonging to adherence clubs had cards with the word *“club”* written on them which meant a much quicker system for drug collection as a CHW explains:

*“They just come with their cards and go straight to where they will get their ARVs pre-packed so that a person doesn’t spend much time in the clinic. These are the people who take their treatment well and they are virally suppressed so they are in clubs*” (CHW_W_48_S).

Any strategy that reduced waiting times for PLHIV were considered as minimising the risk of PLHIV being identified. In South Africa, adherence club initiatives reduced clinic contact and sometimes provided a building where ARVs could be collected and time spent with other clients. Introducing an appointment system that gave PLHIV an appointment time (as opposed to a day) in one clinic was said to have indirectly also addressed stigma by reducing time spent at the clinic. In both countries, CHiPs assisting new clients through processes at the clinic was considered to reduce client time spent waiting in different spaces.

Alterations to demarcated structures were proposed by Zambian participants to allow the clients more privacy accessing demarcated services. One HFW suggested “*sheltered waiting spaces or rooms where no one would see them’ (Z)*, a CHiP proposed *‘PLHIV should have their own pharmacy” (Z)* while, similarly, a CHiP proposed that:

*“The enrolment room should be separate from public areas in order to make people access health services as many do not want to be seen and known that they are HIV positive” (CHiP_W_41_Z).*

Small alterations were suggested in Zambia including turning the benches around in the ART department to face the opposite direction or to construct “*sheltered waiting spaces or room where no one would see them*” for adolescents living with HIV (HFW_W_35_Z). Indeed, in Zambia adolescents were considered to be a key group that could benefit from changes to the spatial organisation of clinics. Demarcated youth services already exist or are being introduced in some clinics with the intention of making young people more comfortable and separating them from the general waiting area where they fear being seen. One South African CHiP also wondered if men who have sex with men should be provided with their own clinic, and some health workers LHIV participants also felt that they should have their own clinic. These suggestions were contrary to the suggestions of other participants that integrated services would reduce identification of PLHIV.

### 2.18 Contesting stigma

There were limited strategies used in the 21 clinics to contest and push against stigma including the use of and changes to spatial organisation, items and the use of policies and guidelines. For example, a common practice for clinic managers in many South African clinics was to routinely address suggestions and complaints raised by clients through the suggestion boxes. In Zambia, a health worker in one clinic recognised the potential of suggestion boxes in combatting stigma:

*“I think it is important because if they [PLHIV] feel segregated or stigmatized, they should be able to write something up and without being known because if someone is affected sometimes they don’t want to be known so you can easily write something and put off in the complaints box and then we will pick it up. I feel if something is picked on people living with HIV something can be discussed in the monthly meeting and we should be able to address it” (HFW_W_57_Z)*

Clinic structural extensions and changes that created either more space and/or privacy were proposed as a possible remedy to stigma. Participants in both countries echoed these sentiments. In Zambia, a CHW proposed that: “*they should create a separate room for triage for people to be free to talk to the nurses”,* while a CHiP in South Africa contended that people's feelings of discomfort could be curtailed if: “*there was more space in the clinic*”.

### 2.19 Limitations of data

This study had some limitations. We were usually only able to interview one participant for each health worker category in each place for each set of interviews. There is a possibility we may have found somewhat different responses if the sample size was larger. This limitation is offset by the range of participants across health workers categories and 21 health facilities across two countries. Only health workers were interviewed, and although they drew on their experiences as well as their perceptions, and although some of them were living with HIV, interviews with a wider group of PLHIV may have broadened the findings. We chose not to do this partly because other literature has established clinic spatial organisation and items as influencing stigma for PLHIV (Gilbert and Walker, 2009, Horter et al., 2017, Raveis et al., 1998, Wringe et al., 2009) and partly because this study was funded to focus on health workers and their experiences.

Another potential limitation is the quality of data collected by the research assistants, which varied across health facilities and countries, although overall the data were considered representative and qualitatively robust. The data collected from South Africa was not as detailed as the data collected in Zambia and more health workers in Zambia disclosed living with HIV compared to South Africa. Furthermore, some participants complained about the length of the interviews and participants often asked for incentives. Due to financial and ethical implications for the wider trial, we were not able to give incentives or tokens of appreciation to these participants.

We were unable to rule out bias in responses given by health workers because interviews were conducted in health facilities, which sometimes made participants cautious and protective of their health facilities (for example, reluctant to relay if PLHIV were ‘talked badly about’). Another bias is that trial staff (CHiPs) may have found it hard to be critical of the HPTN 071 (PopART) trial specific structures and processes. For example, the information desks in Zambia were not said to be linked to stigma through the identification of PLHIV, although trial yellow referral slips were.

## 3 Discussion

In the 21 health facilities in Zambia and South Africa, HIV services were mostly provided in demarcated structures and spaces, usually developed with the aid of global funding and within the confines of the clinic site. Client flow was generally distinctive for PLHIV, either from the clinic entrance or from the reception point, with a few exceptions. According to health worker participants, identification of PLHIV clients was either heightened or diminished by the spatial organisation and items associated with HIV services, including filtering, flow and waiting times, spatial orientation (of buildings, corridors, entry points and waiting areas), inter-connections with the main entrance, openness of waiting areas and registry, and distinctiveness of certain HIV service items (appearance and storage). These features combined with the social identity of PLHIV, time on treatment, relations within clinic spaces and wider community stigma to influence degrees of comfort and discomfort in different clinic spaces. Although demarcated HIV services were linked to the risk of identification and thereby stigma, they had other advantages including client opportunities to share similar experiences and encounter more specialised and friendly staff. More general waiting areas and other services were used by a wider range of clients. These general spaces both allowed PLHIV a degree of anonymity and increased the potential implications of being identified as LHIV, often prompted by ill health, items and public dialogue of staff. These spaces were associated with more blatant gossip and name calling. There were more examples of how to circumvent both contact with services and identification accessing services, than examples of directly challenging negative attitudes and behaviour towards PLHIV. HIV guidelines and policies were far more present in South Africa but not often linked by participants, in either country, to making a space more comfortable.

Both earlier and parallel research in the 21 population catchment areas of the health facilities had established PLHIV fears about “being seen” accessing the facilities (Bond et al., 2016) and how this and internalised stigma contributed to delayed linkage to care (Sabapathy et al., 2017, Seeley et al., 2018), as well as evidence of worrying levels of experienced stigma amongst PLHIV and community members (Hargreaves et al., 2018). These findings about links between anticipated (Earnshaw and Chaudoir, 2009) and internalised stigma (Goffman, 1963) and contact with health facilities in the need to access HIV care and treatment resound with other research in the region (Horter et al., 2017, Sullivan, 2012, Topp et al., 2012, Uebel et al., 2013, Wolf et al., 2014, Wringe et al., 2009). PLHIV have been reported to avoid seeking care due to anticipated and internalised stigma (Hasan et al., 2012, Raveis et al., 1998, Sabapathy et al., 2017, Wringe et al., 2009).

Our research concurs with Sullivan (2012) and Topp et al. (2012) who have also demonstrated how the history of HIV, including the unprecedented scale of the epidemic and global strategies and funds, have structured HIV service delivery around demarcated spaces and distinctive processes (Sullivan, 2012, Topp et al., 2012). The resulting delivery of difference has had to often work within the constraints of existing sites and connect to established infrastructure. Unintentionally, this HIV service delivery has facilitated the identification of PLHIV through creating different and idiosyncratic spatial organisation and items. Because of internalised and wider community stigma, and the accompanying social risk of negative assumptions, PLHIV approach and move through the health facility trying to offset the risk of identification with their need for the benefits of HIV care and treatment. Perhaps inevitably they walk a tightrope of mixed experiences, meeting comfort and discomfort, safety and risk, privacy and exposure.

Providing PLHIV with demarcated services undoubtedly signified social and physical separation at superficial and deeper levels but it could also be both efficient and supportive. This ambivalence was reflected in other research in South Africa where the integration of HIV services was desirable, in part, to reduce stigma of identification, yet both nurses and clients wished for specialised services to develop both expertise, appropriate care and nurse-client relations (Uebel et al., 2013). This inherent contradiction was strikingly apparent in our findings; HIV service spaces were often assessed both comfortable and uncomfortable for a set of core reasons. These reasons included comfort being induced by friendly, supportive and informative relations (between clients, and between staff and clients) and access to care and treatment once within the service spaces. Whereas discomfort was incited by the quality of the space itself (including shortage of space and overcrowding), extended time in the space and the visibility of space, access and items. “Being seen” collecting and having ARVs, for example, was widely reported as being difficult for PLHIV, as noted in other countries (Horter et al., 2017, National Centre in HIV Research, 2012, Nyblade et al., 2009, Wringe et al., 2009).

To add to the complexity of the contrary response to demarcated services, there was a mixed response to most general services and waiting areas. Deemed comfortable because of PLHIV being mixed with other clients, this was offset by the uncomfortable ramifications of anything or anyone signifying HIV status to a more diverse group of clients. In these general spaces, enacted stigma was anticipated more likely than within demarcated spaces, partly due to staff being alleged to be less careful with and supportive to PLHIV. In these general service spaces therefore, the pressure to hide one's status and the risk of unwanted disclosure is greater, and this heightens internalised and anticipated stigma. There were two examples of integrated services (one in Zambia, one in South Africa), and participants in these clinics said this integration reduced identification of PLHIV and stigma, a finding corroborated by other research in Zambia (Topp et al., 2012). Yet the ambivalence about PLHIV being in general service and waiting areas in our analysis warns us that integrating services will not on its own do enough about reducing stigma in this setting.

‘The dualistic nature of place’ (Malpas, 2003 as cited in Sullivan (2012, p.2) partly helps understand why the same clinic space and/or service can be assessed as both comfortable and uncomfortable. He explains how the ‘localised place’ (i.e. the clinic) as well as ‘the imposition of space’ intersect. Sullivan (2012) also proposes that health facilities can be thought of as physical places which encompass overlays of interconnected spaces. Thus, demarcated HIV services are physically separate and can be hard for PLHIV (carrying the ‘space’ of internalised and anticipated stigma linked to the risk of identification, stacked with other abstract identity meanings) to reach discretely. However, our respondents said that once within the demarcated service, if privacy was provided and relations with staff and other clients were supportive, the “space” of this privacy and solidarity pushed back against the risk of being seen accessing the service. Conversely, if the demarcated service within was too outward looking and exposed (for example, the waiting area was open), then the “space” of stigma would continue to weigh heavily. Thus, comfort or discomfort is rarely determined by one factor and it is the combination of the spatial organisation and items with the abstract elements of an environment (Escobar, 2001, Kelly, 2003), including significantly wider community stigma, that makes being seen in health facilities matter.

Gagnon (2015) further reminds us that service delivery is also shaped by more or less liberal politics and policies, which can reinterpret and apply the same procedures in a different way. This might partly explain some divergent responses across Zambia and South Africa. For example, South African participants were more critical of demarcation and quicker to recognise and label discrimination. Given the history of apartheid and deep-rooted segregation, this response is understandable.

Pragmatically, making fundamental changes to some aspects of spatial organisation might not be possible. However, our research pinpoints what was or could be done. When developing or adjusting HIV infrastructure, we could learn from structural interventions in mental health services where approaches to reduce stigma placed the involvement of staff-based perceptions of space and client-based design (Novotná et al., 2011) and dignity, intimacy, humanisation and security (Bil, 2016) at the centre of building design. Our health worker participants have pointed out that HIV services, if demarcated, offer PLHIV more privacy if entry and access is less visible (for example, located at the back of the main clinic), orientated inward (for example, benches placed facing towards the building), and the spaces within are not too compressed and allow for both one on one consultation and the opportunity to interact with other clients LHIV. Enacting these ideas about spatial organisational adjustments to HIV services might improve PLHIV experiences in health facilities. Space arranged in particular ways (referred to as ‘posh design’ by Novotná et al. (2011)), and orientating places and items within them more sensitively (Bil, 2016) was effective at reducing mental health stigma.

Filtering PLHIV clients through a general reception process, and then to demarcated services, could reduce identification but needed to be accompanied by removing HIV identifiers within this filtering system. Indeed, adjustments to HIV items, for example generic files and cards, can also reduce the identification of PLHIV. One South African CHiP advocated for changing the colour coding of PLHIV folders to address stigma. For the same reason, many Zambian health facilities provided a bin next to the pharmacy to dispose of ARV packaging.

Bringing policies and guidelines more visibly, comprehensively and uniformly into practice and reflecting on the design of current health facility audit and People Living with HIV/AIDS friendly checklists could be one way of providing a more comfortable environment for PLHIV and staff especially given the emphasis on this as a stigma reduction strategy.

Integration of services could also reduce identification of PLHIV, but any merging of services needs to still accommodate the need for solidarity and sensitive staff, as well as PLHIV who seek additional privacy (MSM or Health Workers living with HIV). Initiatives for sex workers in Uganda and Zimbabwe (Mbonye et al., 2013, Mtetwa et al., 2013) have shown the value of protected and specially designed space for certain groups of clients.

One effective way of managing stigma is to circumvent it, as demonstrated in this research. Reducing time spent at health facilities through faster flow systems (providing more staffing and fewer steps and more resources) and ART delivery away from the facility (through adherence clubs or community delivery models) would also help partly address the risk of”being seen”, although PLHIV need some contact with clinical services to manage HIV.

Yet avoiding stigma does not confront it. More specific stigma reduction initiatives are needed to address stigma within health facilities and without. Nyblade et al. (2018) are piloting a total health facility approach to reduce stigma. Our findings that health workers in general services are reported to be less careful about identifying PLHIV suggests that this total facility approach is needed. Internalised stigma also needs addressing, building on solidarity experiences and approaches and other stigma tools for health care facilities (Kidd et al., 2015). Wider community stigma reduction programmes might also push back on the risk of “being seen”.

## 4 Conclusion

Health worker participants conveyed the relationship between the spatial organisation and items of HIV stigma as a process of PLHIV navigating access to specialised care and treatment alongside both the potential of being identified by others through contact with HIV specific structures, items, policies and staff and the social risk of unwanted disclosure of HIV status.

Being on ART involves a life-long commitment to frequent contact with clinic services. Yet HIV service encounters within health facilities are laden with opportunities for identification of PLHIV and subsequent unwanted disclosure to others (Gilbert and Walker, 2009, Horter et al., 2017, Raveis et al., 1998, Uebel et al., 2013, Wringe et al., 2009). The process of approaching demarcated HIV services can be perilous for PLHIV, but once inside the services, the experience is often of solidarity with others like you and compassionate care. The ramifications of disclosure, of “being seen” accessing HIV treatment, include being labelled ‘sexually deviant’ or ‘immoral’, being gossiped about and/or ostracized by friends and family, and can lead to decisions not to access care (Hasan et al., 2012, Nyblade et al., 2009).

Given how often HIV programmes are accompanied by changes to health facility structure and processes (Sullivan, 2012, Topp et al., 2012), greater care should be taken to reflect on the spatial organisation and items of facilities and how the use of place intersects with prevailing assumptions about HIV and PLHIV. Across the 21 health facilities there was a delivery of difference for HIV services that heightened the identification of PLHIV within demarcated services and general services and complicated PLHIV navigation of health facility space. There were strategies developed by PLHIV and initiatives by health workers to reduce the chance of “being seen”. Many of these were pragmatic and often physical, spatial or material adjustments to avoid stigma, rather than efforts to directly challenge stigma.

Whilst advocating both pragmatism and organisational adjustments informed by clients and health workers, stigma needs to be confronted more directly and unambiguously. Addressing internalised stigma and specific anti-stigma education in health facilities with health workers are the most promising approaches for truly shifting and tackling stigma. Until this happens, stigma outside health facilities will continue to be carried within, intersecting with specific structures, items, policies and staff.

## Footnotes

1 Participants are identified by: type of health worker –health facility worker (HFW), community health worker (CHW) or Community HIV-care provider (CHiP); gender – w (woman) or m (man); age – in years; and country – Z (Zambia), S (South Africa). Community names or numbers are not included to avoid risk of identification.
